# Activated PPARγ Abrogates Misprocessing of Amyloid Precursor Protein, Tau Missorting and Synaptotoxicity

**DOI:** 10.3389/fncel.2019.00239

**Published:** 2019-06-12

**Authors:** Susanne Moosecker, Patrícia Gomes, Chrysoula Dioli, Shuang Yu, Ioannis Sotiropoulos, Osborne F. X. Almeida

**Affiliations:** ^1^Department Stress Neurobiology and Neurogenetics, Max Planck Institute of Psychiatry, Munich, Germany; ^2^Graduate School, Technical University of Munich (TUM), Munich, Germany; ^3^Life and Health Sciences Research Institute (ICVS), Medical School, University of Minho, Braga, Portugal; ^4^ICVS/3B’s – PT Government Associate Laboratory, Guimarães, Portugal

**Keywords:** Alzheimer’s disease, amyloid beta, pioglitazone, PPARγ, neurons, Tau missorting, synaptic degradation

## Abstract

Type 2 diabetes increases the risk for dementia, including Alzheimer’s disease (AD). Pioglitazone (Pio), a pharmacological agonist of the peroxisome proliferator-activated receptor γ (PPARγ), improves insulin sensitivity and has been suggested to have potential in the management of AD symptoms, albeit through mostly unknown mechanisms. We here investigated the potential of Pio to counter synaptic malfunction and loss, a characteristic of AD pathology and its accompanying cognitive deficits. Results from experiments on primary mouse neuronal cultures and a human neural cell line (SH-SY5Y) show that Pio treatment attenuates amyloid β (Aβ)-triggered the pathological (mis-) processing of amyloid precursor protein (APP) and inhibits Aβ-induced accumulation and hyperphosphorylation of Tau. These events are accompanied by increased glutamatergic receptor 2B subunit (GluN2B) levels that are causally linked with neuronal death. Further, Pio treatment blocks Aβ-triggered missorting of hyperphosphorylated Tau to synapses and the subsequent loss of PSD95-positive synapses. These latter effects of Pio are PPARγ-mediated since they are blocked in the presence of GW9662, a selective PPARγ inhibitor. Collectively, these data show that activated PPARγ buffer neurons against APP misprocessing, Tau hyperphosphorylation and its missorting to synapses and subsequently, synaptic loss. These first insights into the mechanisms through which PPARγ influences synaptic loss make a case for further exploration of the potential usefulness of PPARγ agonists in the prevention and treatment of synaptic pathology in AD.

## Introduction

While age represents the greatest risk for developing AD, modern lifestyle which frequently leads to obesity and T2D also appears to increase risk for developing AD (Biessel and Despa, 2018). While the link between T2D and AD may be equivocal (see Vemuri et al., 2017) and awaits longitudinal studies in humans that use new imaging technologies such as positron emission tomography (PET; see Arnold et al., 2018), evidence exists for an association between perturbed insulin signaling and AD histopathology, namely, Aβ aggregates and Tau protein-containing neurofibrillary tangles (NFT) (de la Monte and Tong, 2014; Mullins et al., 2017; Arnold et al., 2018). The latter, complement reports that animal models of diabetes exhibit cognitive impairments and features of AD neuropathology (Clodfelder-Miller et al., 2006). Together, these data make it plausible that AD may be amenable to antidiabetic treatments (for review, see Govindarajulu et al., 2018).

Insulin insensitivity, a mainstay of T2D, can be treated by activating PPARγ with TZD such as Pio (Ahmadian et al., 2013). Various TZD have also been shown to improve cognition in AD patients and mouse models of the disease (Risner et al., 2006; Hanyu et al., 2010; Mandrekar-Colucci et al., 2012; Searcy et al., 2012; Ahmadian et al., 2013). However, mechanisms through which TZD exert their effects in the brain remain elusive. In support of previous reports (Moreno et al., 2004; Lu et al., 2011; Warden et al., 2016), we recently mapped the expression of functional PPARγ in the rodent brain (Pissioti, 2016; Moosecker, 2018) and demonstrated the presence of TZD-responsive substrate(s) in the brain. Among other brain areas, PPARγ were localized in the frontal cortex and hippocampus, two cognitive centers that are particularly susceptible to neurodegeneration in AD.

In contrast to earlier views that aggregated Aβ and Tau are responsible for cognitive dysfunction in AD, recent work implicates soluble Aβ and hyperphosphorylated Tau protein as triggers of synaptic dysfunction and loss. Since synaptic malfunction and loss correlates strongly with cognitive deficits in AD patients (Wang and Mandelkow, 2016; Müller et al., 2017), a better understanding of the pathophysiology of soluble Aβ and hyperphosphorylated Tau is imperative. While our own early studies showed that soluble Aβ causes synaptic degradation (Roselli et al., 2005, 2011; Liu et al., 2010; Chang et al., 2016), the role of Tau and its abnormal hyperphosphorylation in synaptic dysfunction is gaining increasing attention (Kimura et al., 2007; Ittner et al., 2010; Lopes et al., 2016). An important impetus for this shift in focus was the demonstration that Tau, usually considered to be an axonal protein (Kubo et al., 2018), is localized in dendrites (Hoover et al., 2010; Ittner et al., 2010) and that, in fact, Tau can be *de novo* synthesized and hyperphosphorylated in dendrites and spines (Frandemiche et al., 2014; Pinheiro et al., 2016; Kobayashi et al., 2017).

The *in vitro* studies reported here focused on whether, and how, activation of PPARγ can influence the synaptotoxic effects of Aβ and/or Tau. Our experiments show that Pio-activated neuronal PPARγ inhibits APP misprocessing and protects against Aβ-induced synaptic degradation. In addition, the PPARγ agonist attenuated Tau missorting and hyperphosphorylation in Aβ-exposed neurons.

## Materials and Methods

### Drugs

Soluble Aβ_1–42_ was prepared from peptide obtained from American Peptide Co. (Sunnyvale, CA, United States; Cat. #62-0-80), according to Roselli et al. (2005) and Stine et al. (2011) and used at a concentration of 1 μM. Pio (Pio, 10 μM) and GW9662 (1 μM), both purchased from Sigma-Aldrich (Taufkirchen, Germany) were used after solution in dimethylsulfoxide (DMSO; final DMSO concentration 0.01%). Doses of Pio were chosen on the basis of previous cell culture studies (Inestrosa et al., 2004; Chang et al., 2015).

### Cell Culture

The human neuroblastoma cell line, SH-SY5Y [American Tissue Culture Collection (ATCC^®^), CRL-2266^TM^, Germany] was cultured in Minimum Essential Medium with Glutamax^®^, supplemented with 10% FCS, 1% penicillin-streptomycin, and 2 mM L-glutamine. When ∼ 20% confluent (6 × 5^10^ cells/well of a 6-well plate), cells were differentiated with 50 μM retinoic acid (Sigma-Aldrich) in 1% FCS medium for 5 days, followed by 20 ng/ml nerve growth factor (NGF; Bio-Techne, Wiesbaden, Germany) for a further 5 days. Cells were maintained at 37°C in an incubator with 5% CO_2_ and 95% relative humidity.

### Primary Neural Cell Cultures

Primary frontocortical and hippocampal cultures were prepared from brains of CD1 mice aged 5 days, according to previously described protocols (Lu et al., 2005; Roselli et al., 2005). For molecular/biochemical analyses, cells were plated on gelatine/poly-D-lysine-coated plates and maintained in Neurobasal/B27 medium supplemented with basic fibroblast growth factor (10 ng/ml; Life Technologies (Eggenstein, Germany) and kanamycin (100 μg/ml; Life Technologies). For immunocytochemical analyses, cells were plated at a density of 400–500 cells/mm^2^ on poly-D-lysine-coated glass coverslips (Yu et al., 2010), and grown in Neurobasal/B27 before use after 14 days *in vitro* (14 DIV). Cultures were comprised of 15–20% of mature [microtubule-associated protein 2 (MAP2)-positive] neurons, 20–25% of astrocytes [glial acidic fibrillary acidic protein (GFAP)-positive], and ∼1% of O4-positive oligodendrocytes; microglia (anti-CD68, -CD1b and -Iba1 labeled) were undetectable. Experiments adhered to European Union Council Directive (2010/63/EU) and local regulations on use of animals.

### Cell Viability Assay

The MTS assay kit (CellTiter 96^®^ AQueous One Solution Cell Proliferation Assay (Promega, Mannheim, Germany) was used to monitor cell viability, following the manufacturer’s instructions. Briefly, after exposure to MTS solution (3 h in dark), the optical density (490 nm) of the supernatant was measured in an ELISA reader (BioTek Instruments, Winooski, VT, United States).

### Immunofluorescence Staining and Image Analysis

Cells were stained as described by Roselli et al. (2005). Briefly, cells were fixed in ice-cold 4% paraformaldehyde for 15 min, washed in PBS (3 × 5 min) before permeabilization with 0.1% Triton X-100 (30 min), and blocked in 10% FCS (30 min, RT). Primary and secondary antibody solutions were prepared in 0.01 M PBS containing 0.1% Triton X-100 and 10% FCS. Cells were incubated (16 h; 4°C), with primary antibodies against postsynaptic density-95 (PSD-95; 1:1000; Neuromab, Davis, CA, United States; #75-028), synapsin 1,2 (1:1500; Synaptic Systems, Göttingen, Germany; #16002), and/or pTau (pS396-Tau) (1:1000; abcam, Cambridge, United Kingdom; ab109390) in PBST (0.01 M PBS + 0.03% Triton X100). After washing (3 × 30min in 0.01 M PBS), cells were incubated with one of the following secondary antibodies, as appropriate: goat-anti-rabbit Alexa Fluor 488 (1:1000; Invitrogen, Eggenstein, Germany; # A110374) or goat-anti- mouse Alexa Fuor 594 (1:1000; Invitrogen; # A110029)] for 1 h at RT; nuclei were counterstained with followed by Hoechst dye 33341 (Sigma; 1:50000; 10 min, RT). Images were obtained using a laser scanning confocal microscope (Olympus Fluoview 1000, Hamburg, Germany). For image quantification, 100 cells in 5 separate fields on each coverslip (3–6 coverslips per condition) were analyzed. The number of stained puncta on a defined dendritic length (100 μm) were quantified using SynPAnal software to monitor synaptic density (Danielson and Lee, 2014).

### Immunoblotting

Cells were homogenized in lysis buffer [10 mM HEPES pH 7.9, 150 mM NaCl, 1 mM EGTA, 10% glycerol, 1% NP-40, Complete Protease Inhibitor (Roche, Mannheim, Germany), Phosphatase Inhibitor Cocktails II and III (Sigma)] using a sonifier (5 pulses, 20 kHz). After centrifugation (14,000 ×*g*; 20 min), the protein content of the lysates (supernatants) were determined by the Lowry assay (Lowry et al., 1951); spectroscopic measurements (absorption wavelength: 750 nM) were made with a Synergy-HT plate reader (BioTek Instruments, Winooski, VT, United States). Sodium dodecyl sulfate-polyacrylamide gel (10%) electrophoresis (SDS-PAGE) was used to resolve heat-denatured (95^o^ C; 10 min) protein lysates (30 μg). After electrophoresis, proteins were transferred onto 0.2 μm nitrocellulose membranes (BioRad, Hercules, CA, United States) by Turbo Transfer (BioRad). Transfer quality was assessed by incubating with Ponceau-S solution. Membranes were subsequently blocked in 5% non-fat milk or 5% BSA in TBS-T (1 h, RT), before overnight incubation (4^o^C) with one of the following primary antisera: APP A4 (1:500; Millipore, Burlington, MA, United States; #MAB348), BACE (D105E5) (1:1000; Cell Signaling; #5606), nicastrin (1:1000; Sigma; #N16660), pS202-Tau (1:1500; Abcam; ab108387); pT205-Tau (1:1500; Abcam; ab4841), pT231-Tau (1:1500; Abcam; ab151559), pS356-Tau (1:1500; Abcam; ab92682), PHF1 (p396/404-Tau; 1:1000; kind gift form Dr. Peter Davies, New York, NY, United States), Tau5 (1:1500; Abcam; ab 80579), GluN2B (1:1000, Abcam 65783), pSer9-GSK3β and total GSK3β (1:1000, Cell Signaling) and either actin (1:2500; Chemicon/Fischer Scientific, Munich, Germany; #MAB1501R) or GAPDH (1:1500, Abcam; ab8245). After thorough washing, membranes were incubated with a corresponding horseradish peroxidase (HRP)-conjugated secondary antibody [goat anti-rabbit (1:1000; Fischer Scientific; #31460) or goat anti-mouse (1:2000; BioRad; 170-6516)] for 1 h (RT). Clarity^TM^ Western ECL reagent (Biorad) was used to visualize (ChemiDoc MP Imaging System; BioRad) and quantify (ImageLab 5.1 Software from BioRad) proteins.

### PCR Analysis

Total RNA was isolated from cell lysates using the NucleoSpin (RNA) kit (Macherey-Nagel, Duren, Germany) and RNA concentrations were determined with a NanoPhotometer (SmartSpec^TM^ Plus, Biorad).

### Reverse Transcription

Complementary DNA (cDNA) was prepared from 1 μg RNA using a RevertAid RT Reverse Transcription kit (Thermo Scientific) with an oligo deoxythymine (dT) primer. Polymerase chain reactions (PCR) were performed using Taq DNA Polymerase kits (Fermentas/ThermoFisher) and the following primers:

*ABCA1*: fwd 5′-GACATCCTGAAGCCAATCC-3′
rev 5′-GTAGTTGTTGTCCTCATACC-3′
*PGC-1α*: fwd 5′-CGTGTCGAGACTCAGTGTC-3′
rev 5′-GTGTCTGTAGTGGCTTGATTC-3′
GAPDH: fwd 5′-CCATCACCATCTTCCAGG-3′
rev 5′-GTTGAAGTCGCAGGAGACAAC-3′

The PCR products were quantified using a Roche LightCycler 96. Relative expression levels of target genes were computed according to Pfaffl (2001).

### Statistical Analysis

Statistical analysis and graphic representations were performed using GraphPad Prisma software (GraphPad, San Diego, United States). Numerical data were analyzed by 1-way ANOVA or Kruskall-Wallis tests, and *post hoc* tests, as appropriate. Values were considered significant when *p* < 0.05.

## Results

### Activation of PPARγ Attenuates APP Misprocessing, Tau Accumulation and Aβ-Induced Neurotoxicity in Differentiated SHSY5Y Cells

Initially, we examined whether differentiated human SH-SY5Y cells express functional PPARγ. For that purpose, we monitored the expression of two PPARγ target genes, *peroxisome proliferator-activated receptor gamma coactivator 1-α (PGC-1*α*)* and *ABCA1* (Strum et al., 2007; Kang and Rivest, 2012) after treating cells with the potent PPARγ agonist, Pio (10 μM; 24 h). As shown in Supplementary Figure 1, Pio induced the expression of the mRNA levels of *PGC-1*α and *ABCA1*. These effects were abolished when cells were co-treated with Pio and the PPARγ antagonist GW9662, indicating that the actions of Pio were mediated by endogenous PPARγ (Supplementary Figure 1).

**FIGURE 1:**
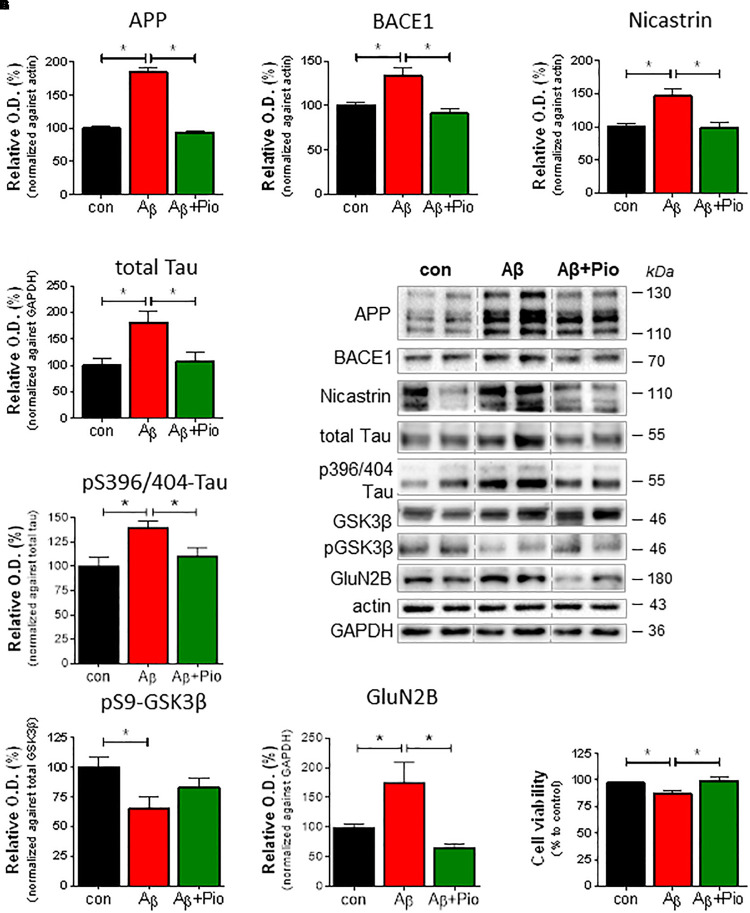
Pioglitazone (Pio) attenuates APP misprocessing, Tau accumulation and Aβ-induced neurotoxicity in differentiated SHSY5Y cells.Exposure to Aβ (1 μM, 24 h) upregulated the levels of APP (*p* < 0.0001) **(A,E)**, BACE-1 (*p* = 0.002) **(B,E)**, and nicastrin (*p* = 0.003) **(C,E)**; co-treatment with the PPARγ agonist, Pio (Pio; 10 μM) abolished these effects of Aβ (*p*_APP_ < 0.0001; *p*_BACE_ = 0.001; *p*_nicastrin_ = 0.038). Exposure to Aβ increased total Tau levels (*p* = 0.002) in a Pio-reversible manner (*p* = 0.006) [1-way ANOVA *F* = 7.745; *p* = 0.001] **(D,E)**. Treatment of cells with Aβ resulted in elevated levels of pS396/404-Tau (*p* = 0.015) in a Pio-reversible manner (*p* = 0.029) [1-way ANOVA *F* = 5.551; *p* = 0.015] **(F,E)**; Aβ also reduced levels of pSer9-GSK3β (inactive GSK3β; (*p* = 0.029) **(G,E)**. Co-treatment of cells with Aβ and Pio failed to show Aβ-driven elevation of GluN2B protein levels (*p* = 0.023) [1-way ANOVA *F* = 7.553; *p* = 0.0027] **(H)** and Aβ-stimulated cell death (*p* = 0.008) [1-way ANOVA *F* = 6.113; *p* = 0.007] **(I)**; *N* = 3-5/condition; different cell cultures. All numeric data represent means ± S.E.M.

The neurodegenerative cascade leading to AD is initiated by Aβ. Here, to examine the role of PPARγ in modulating neuropathological markers of AD, we exploited our previously described model in which Aβ was shown to increase misprocessing of APP (Catania et al., 2009). Western blot analysis revealed that treatment of cells with Aβ (1 μM; 24 h) upregulated APP levels (Figure 1A,E) as well as those of β-secretase (BACE1) (Figure 1B,E) and nicastrin (Figure 1C,E) which sequentially contribute to the generation of Aβ. It may be extrapolated from these observations that exogenous Aβ stimulates the misprocessing of APP into Aβ. Importantly, concomitant exposure with Pio abolished the ability of Aβ to increase the expression of APP, BACE1 and nicastrin protein (Figure 1A–C,E).

Growing appreciation of the neurotoxic role of Tau protein (Takashima et al., 1998; Rapoport et al., 2002; Ittner et al., 2016) prompted an examination of how Aβ, in the presence or absence of Pio (10 μM), influences Tau metabolism in differentiated SH-SY5Y cells. As shown in Figure 1D,E, immunoblot analysis revealed that incubation of cells with Aβ results in increased levels of total Tau, an effect blocked when cells were co-treated with Pio. Further, Aβ treatment increased Tau phosphorylation (pS396/404-Tau) (Figure 1F) while decreasing the amount of inactive (pSer9) GSK-3β (Figure 1G); the effects of Aβ on pTau were also reversed by Pio (Figure 1E–G). In addition, we monitored the levels of NR2B subunit of the glutamate (NMDA) receptor (GluN2B) which is strongly implicated in neurotoxicity. Consistent with previous evidence that GluN2B largely mediate the neurotoxic actions of Aβ and Tau (Ittner et al., 2010), we observed that Aβ elevate GluN2B levels (Figure 1H,E) and, at the same time, compromises cell viability (Figure 1I). Both of these Aβ-induced phenomena were blocked when cells were co-treated with Pio, although Pio *per se* did not have any effect, i.e., Pio did not exert any effect in the absence of Aβ (Supplementary Figure 2).

### Synaptic Degradation Induced by Aβ in Primary Neuronal Cultures Is Blocked by Pio Treatment

**FIGURE 2:**
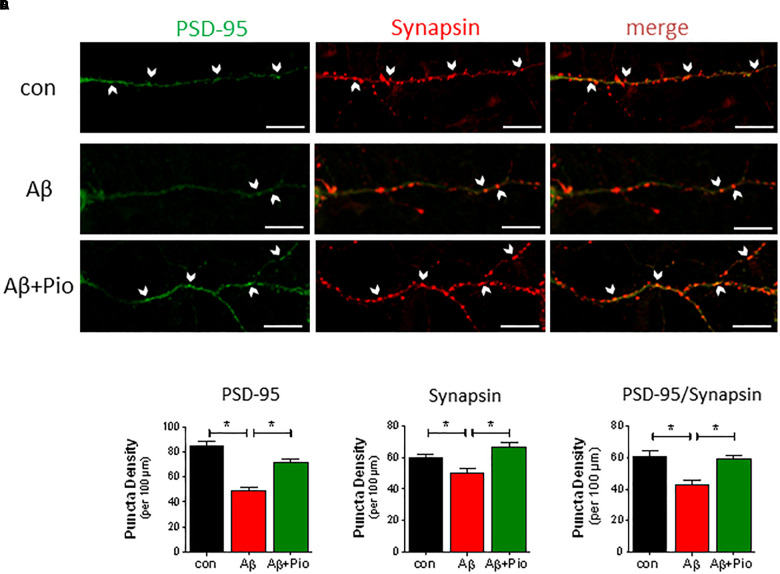
Aβ-induced synaptic loss is rescued by Pio treatment. **(A)** Representative confocal images of synapses (identified by apposed PSD-95-, *green*, and synapsin-*, red*, immunoreactive elements) in mouse primary neuronal cultures under control conditions and exposure to Aβ (1 μM, 24 h) alone Aβ + Pio (Pio, PPARγ agonist). Examples of apposed PSD-95- and synapsin-positive staining are marked with white arrows. **(B)** Puncta analysis of dendrites revealed that Aβ decreases the density of PSD-95 puncta (*p* < 0.0001 vs. control) and that Pio reverts the effects of Aβ (*p* < 0.0001) [1-way ANOVA, *F*_2,63_ = 35.01; *p* < 0.0001]. **(C)** The reduced number of synapsin-positive puncta after Aβ treatment were restored to control levels when cells were co-exposed to Pio (*p* = 0.001) [1-way ANOVA, *F*_2,63_ = 10,68; *p* < 0.0001]. **(D)** Synapses, doubled-labeled for PSD-95 and synapsin immunoreactivity, were reduced after treatment with Aβ (*p* = 0.0003), indicating synaptic degradation and loss. The latter was absent in cells exposed to both Aβ and Pio (*p* = 0.0012) [1-way ANOVA, *F*_2,63_ = 10.32; *p* < 0.0001]; *N* = 15 non-overlapping fields/condition; different cultures, Means ± S.E.M are shown. Scale bar: 10 μm.

Since both Aβ and hyperphosphorylated Tau are known to disrupt synaptic function, we next examined the potential of Pio to prevent Aβ-driven synaptotoxicity in differentiated cultures derived from mouse frontal cortex and hippocampus; cultures were used at DIV 14, when 15–20% of the cells are MAP2-positive (mature neurons) bearing synapsin 1,2-immunoreactive mature synapses. Both frontocortical and hippocampal cultures express transcriptionally active PPARγ and display similar responses to Aβ (Moosecker, 2018). As shown previously (Roselli et al., 2005; Liu et al., 2010), synaptic loss was assessed by counting (SynPal software) apposed postsynaptic PSD-95- and presynaptic synapsin 1, 2- immunoreactive elements in confocal images (Figure 2A).

Neurons exposed to Aβ showed fewer synapses (reduced PSD-95 and synapsin puncta density (Figure 2A–D). This result is consistent with previous findings by Roselli et al., (2005) and Liu et al. (2010). Interestingly, the synaptic loss caused by Aβ was blocked in the presence of the PPARγ agonist Pio (Figure 2A–D), suggesting that Pio protects against Aβ synaptotoxicity.

### Pioglitazone Counteracts Aβ-Driven Tau Hyperphosphorylation in Primary Cultures

**FIGURE 3:**
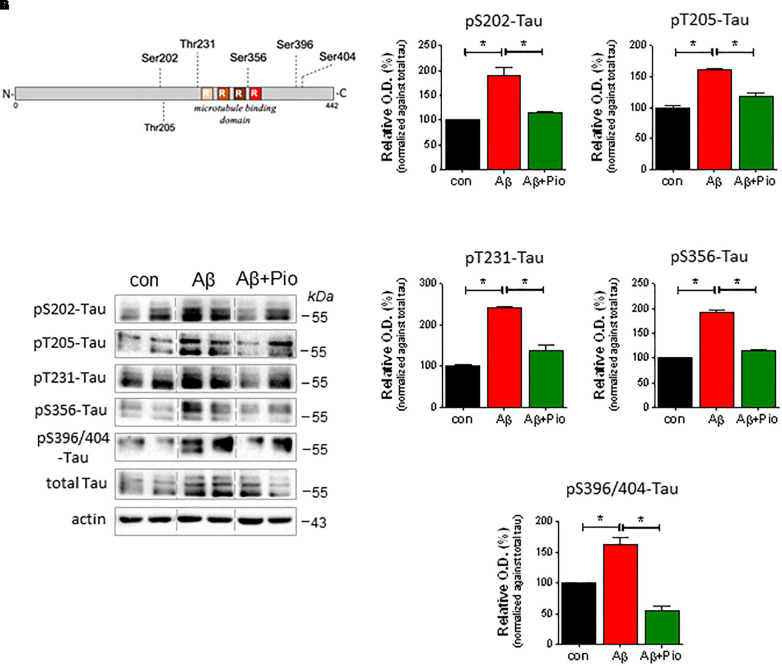
Pioglitazone (Pio) counteracts Aβ-driven Tau hyperphosphorylation. **(A)** Schematic representation of Tau protein and the different phosphorylation epitopes analyzed in this study. (**B–G)** Effects of Aβ (1 μM, 24 h), in presence and absence of Pio (Pio; 10 μM), on the expression of different forms of pTau in 14 DIV primary neurons. Representative immunoblots are shown in **(F)**. Semi-quantitative analysis reveals that, as compared to vehicle treatment, Aβ induces higher levels of pS202-Tau **(B)**, pT205-Tau **(C)**, pT231-Tau **(D)**, pS356-Tau **(E)** and pS396/404-Tau **(G)** (*p* < 0.05 in all cases); all of these effects were attenuated (*p* < 0.05) when cells were co-treated with Pio **(B–G)** [Kruskal-Wallis test *H*_2_ = 9.881, *p* = 0.0002]; *N* = 3-5/condition; different cell cultures. Semi-quantitative data are shown as means ± S.E.M.

In light of the fact that (i) Aβ induces Tau hyperphosphorylation (Takashima et al., 1998; Sotiropoulos et al., 2011), (ii) Tau hyperphosphorylation is causally linked to synaptic dysfunction and loss (Kimura et al., 2007), and (iii) Pio reduces the cytotoxic actions of Aβ (cf. Figure 1J), we also examined whether Pio can interfere with Aβ-triggered Tau hyperphosphorylation by monitoring drug effects on different phospho-epitopes of Tau (around and within its microtubule-binding domain) (see Figure 3A).

Our analysis revealed that exposure of primary neurons to Aβ leads to increased levels of various forms of pTau (pSer202-Tau, pThr205-Tau, pThr231-Tau, pSer356 and pSer396/Ser404), as shown in Figure 3F (representative immunoblots) and Figure 3B–G (semi-quantitative data). Notably, although Pio alone did not exert any effect on Tau protein (Supplementary Figure 2), the PPARγ agonist blocked Aβ-upregulated levels of pSer202-Tau, pThr205-Tau, pThr231-Tau, pSer356 and pSer396/Ser404-Tau (Figure 3A–G).

### Prevention of Aβ-Induced Synaptic Missorting of Tau by Pio

Whereas Tau protein expression is confined to axons under normal conditions (Kubo et al., 2018), increased levels of Aβ have been shown to re-direct Tau into dendrites and dendritic spines, via a process termed “missorting” which triggers synaptic loss (Ittner et al., 2010; Zempel et al., 2010). Having observed that Pio exhibits a protective effect against Tau hyperphosphorylation (see Figure 3), we next monitored the effect of Pio on the localization of pTau (specifically, pSer396-Tau) in dendrites and synapses.

**FIGURE 4:**
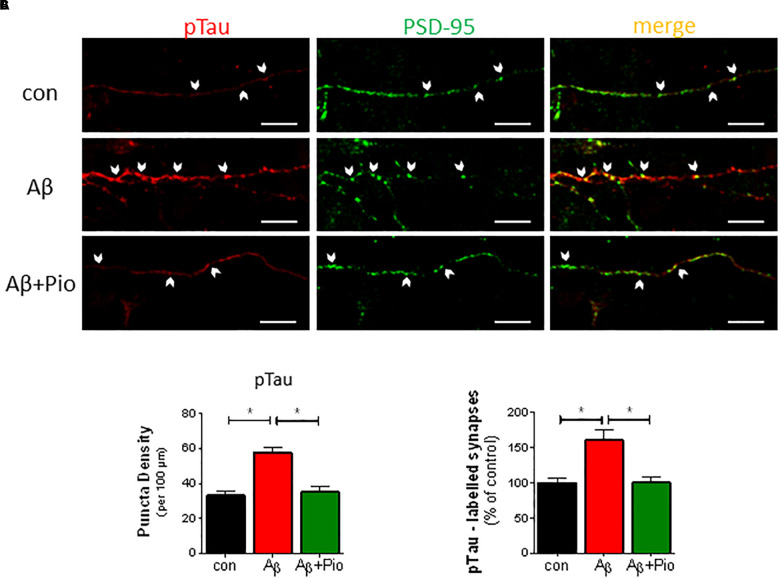
Prevention of Aβ-driven synaptic missorting of phospho-Tau by Pio. **(A)** Representative immunofluorescent images of 14 DIV neurons stained with antibodies against pTau (pSer396-Tau, *red*) and PSD-95 (*green*) after treatment with Aβ (1 μM, 24 h) and/or Pio (10 μM). Examples of apposed PSD-95- and pTau-positive staining are marked with white arrows. **(B)** Density of pTau-positive puncta in dendrites increases after exposure to Aβ (*p* < 0.0001 vs. control) in a Pio-reversible fashion (*p* = 0.0002 vs. Aβ alone) [1-way ANOVA, *F*_2,44_ = 18.02; *p* < 0.0001]. **(C)** The percentage of PSD-95-labeled synaptic puncta that are pTau-positive is higher in Aβ- vs. vehicle-treated cells (*p* = 0.0002), indicating missorting of pTau to synapses. In the presence of Pio, Aβ exhibited a weaker effect on pTau missorting (*p* = 0.002) [1-way ANOVA, *F*_2,76_ = 11.79; *p* < 0.0001]; *N* = 15 non-overlapping fields/condition; different cultures. Means ± S.E.M. are depicted. Scale bar: 10 μm.

As shown in Figure 4, treatment of neuronal cultures with Aβ was found to increase p-Tau immunoreactivity in dendritic puncta (Figure 4A,B). The latter was abrogated when cultures were simultaneously exposed to Pio (Figure 4A,B). More detailed analysis of the data, in which the number of PSD-95/synapsin-positive synapses labeled with pTau were quantified, confirmed that Pio can effectively prevent the Aβ-induced mislocalization of pTau in PSD-95/synapsin-positive puncta (Figure 4C). Note that, Pio itself did not display activity on any of the synaptic parameters monitored (Supplementary Figure 3).

**FIGURE 5:**
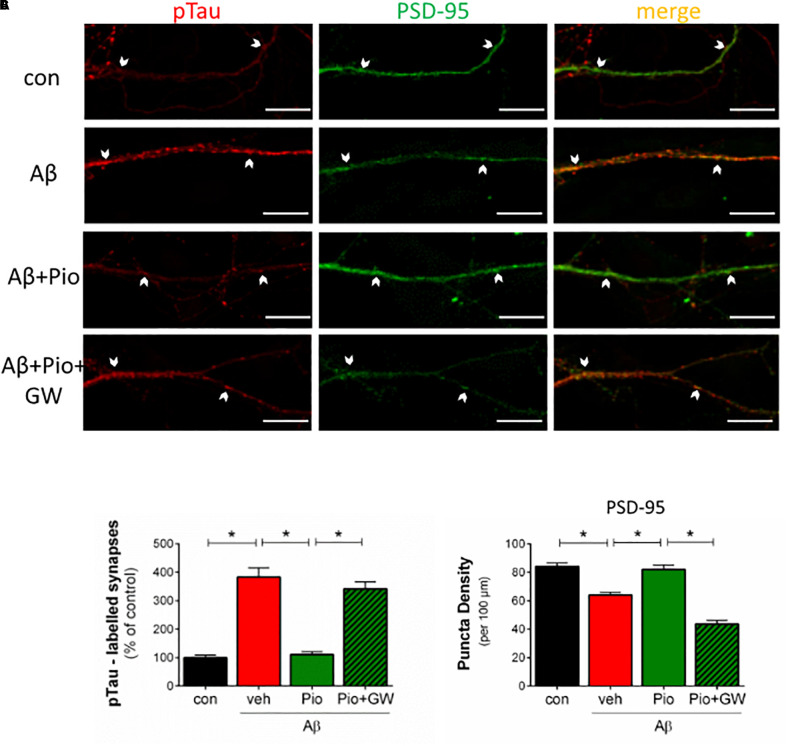
Protective actions of Pio against Aβ-induced missorting of Tau and synaptic loss are PPARγ-mediated. **(A)** Primary cultures were pre-treated with the specific PPARγ antagonist GW9662 (GW) before exposure to Aβ alone, or Aβ + Pio (PPARγ agonist). Cells were immunostained for PSD-95 (*green*) and pTau (pSer396-Tau; *red*) and examined by confocal microscopy. Examples of apposed PSD-95- and pTau-positive staining are marked with white arrows. **(B)** Shows that Aβ leads to an increase (*p* < 0.0001) in the percentage of pTau-positive synaptic puncta (PSD-labeled), an effect blocked when Pio is also present. Importantly, Pio failed to counter the effects of Aβ when cells were pre-exposed to GW9662 (*p* < 0.0001 in all cases) [1-way ANOVA, *F*_3,37_ = 62.40; *p* < 0.0001]. **(C)** Shows that GW9662 also blocks the protective effects of Pio (*p* < 0.0001) against the loss of PSD-95-positive puncta in neurons treated with Aβ (*p* = 0.0002) [1-way ANOVA, *F*_3,48_ = 45.44; *p* < 0.0001]; *N* = 15 non-overlapping fields/condition. Numerical data shown represent means ± S.E.M. Scale bar: 10 μm.

Since the results described in Figure 1–4 clearly indicate involvement of PPARγ in regulating APP and Tau metabolism, as well as neuronal survival, we considered it important to investigate whether the interruption of Aβ-induced synaptic loss and mislocalization of pTau by Pio depends on PPARγ. To this end, primary neurons were pre-treated with the PPARγ antagonist GW9662 before exposure to Pio + Aβ. Indeed, as shown in Figure 5A–C, PPARγ were demonstrated to mediate the reversal of Aβ-induced missorting of Tau to dendritic spines by Pio: the protective potency of Pio was lost in GW9662-treated cells. Briefly, the percentage of pTau-labeled synapses was greater in Pio + Aβ-treated cells than in cells receiving the combination of GW9662, Pio and Aβ (Figure 5B). Similarly, on the basis of PSD-95 puncta density measurements, GW9662 was found to neutralize the rescuing effect of Pio on Aβ-driven synaptic loss (Figure 5C). Together, this set of data demonstrates that PPARγ mediate the rescuing actions of Pio against Aβ-triggered synaptotoxity by preventing the missorting of Tau to synapses.

## Discussion

Pioglitazone, a pharmacological agonist of the nuclear receptor PPARγ, acts as an insulin sensitizer and is used to treat T2D, a risk factor for AD (Ahmadian et al., 2013; Arnold et al., 2018; Biessel and Despa, 2018). Reports that Pio and other TZD improve cognitive performance in AD patients and in mouse models of the disease (Risner et al., 2006; Hanyu et al., 2010; Mandrekar-Colucci et al., 2012; Searcy et al., 2012; Ahmadian et al., 2013) suggest that PPARγ may be potential therapeutic targets in AD (Zolezzi et al., 2017). A central question in the present work was whether the pro-cognitive and anti-neurodegenerative effects of Pio reflect direct actions in the brain or if they represent a collateral benefit of improved insulin sensitivity. Notably, some authors have also attributed the neuroprotective effects of TZD to their anti-inflammatory and/or -oxidative properties (Heneka et al., 2005, 2015; Wang X.K. et al., 2017; Zhang et al., 2017), phenomena that could help explain the purported role of activated PPARγ in a spectrum of neurological diseases (Zolezzi et al., 2017).

Complementing data reported by Xu et al. (2014), we here show that Pio counteracts the detrimental effects of Aβ on neural cell viability. In addition, we found that Pio dampens Aβ-stimulated misprocessing of endogenous APP into Aβ through the mediation of β-secretase 1 (BACE1) and γ-secretase (see Catania et al., 2009). These observations are consistent with previous reports that TZD can reduce APP misprocessing as well as Aβ deposition in transgenic mouse models of AD (Escribano et al., 2010; Mandrekar-Colucci et al., 2012; Skerrett et al., 2015) as well as in primary neural cultures (Mandrekar-Colucci et al., 2012; Skerrett et al., 2015) and in neural cell lines overexpressing APP (Camacho et al., 2004). The inhibitory effect of Pio on APP misprocessing likely reflects transcriptional regulation of BACE1 by PPARγ: the *BACE1* promoter harbors a PPARγ response element (PPRE) (Heneka et al., 2005; Sastre et al., 2006; Chen et al., 2009; Wang X. et al., 2017) and genetic deletion of related PPAR isoforms (PPAR β/δ) leads to increased BACE1 expression in mice (Barroso et al., 2013).

We also report here that Pio prevents the ability of exogenous Aβ to increase the expression of Tau and several phospho-Tau epitopes that are found in the brains of AD patients (see Iqbal et al., 2016). The latter observation, which is consistent with a previous report that TZD reduce Tau hyperphosphorylation in a 3 × Tg mouse model of AD (Yu et al., 2015), is important since Tau is now recognized as a critical mediator of the synaptotoxic effects of Aβ (Kimura et al., 2007; Roberson et al., 2007; Ittner et al., 2010; Lopes et al., 2016). Together, this and the previous set of data suggest that activation of PPARγ breaks the link between Aβ-induced neurotoxicity and tau pathology.

The present work also examined the possibility that PPARγ may be involved in synaptic dysfunction and loss, two events that appear to underpin memory loss in AD patients (Xu et al., 2014; Chen et al., 2015; Canter et al., 2016). In line with our previous findings in frontocortical (Roselli et al., 2005) and hippocampal (Liu et al., 2010) neurons, Aβ was here found to induce a loss of synapses, seen as a reduction in the number of apposed synapsin- and PSD-95-immunoreactive puncta. Further, and in confirmation of results reported by Xu et al. (2014), we show that Aβ-induced degradation of synapses can be blocked by co-treating neurons with Pio. Interestingly, earlier studies reported that TZD-activated PPARγ promote synaptic plasticity (Nenov et al., 2015). Notwithstanding a role for neurotrophins in mediating the neuroplastic effects of activated PPARγ (Kariharan et al., 2015), analysis of the data presented in this paper suggest, for the first time, that PPARγ-mediated inhibition of Tau mislocalization (missorting) to the dendritic compartment represents an important mechanism through which TZD impede progression of the neurodegenerative cascade initiated by Aβ. In agreement with earlier reports (Hoover et al., 2010; Zempel et al., 2010), we observed that Aβ leads to an accumulation of hyperphosphorylated Tau in dendritic spines; the latter event is believed to activate a pathway that upregulates GluN2B receptor expression which, in turn, culminates in synaptic dysfunction and elimination (Hoover et al., 2010; Ittner et al., 2010; Lopes et al., 2016). Briefly, our results indicate that Pio prevents Aβ-driven hyperphosphorylation and intraneuronal trafficking of Tau. It is important to note here that Hoover et al. (2010) demonstrated that hyperphosphorylation of Tau is necessary for the missorting and accumulation of Tau at synapses.

In summary, the current experiments provide new insights into the mechanisms through which activated PPARγ can provide neuroprotection by acting directly on neural substrates, independently of their insulin-sensitizing properties in the periphery. Interestingly, the protective actions of Pio only became manifest when neurons were challenged with an insult, namely, elevated Aβ levels. Lastly, this work introduces the notion that prevention of the mislocalization of Tau to dendrites is a key mechanism underlying the neuroprotective actions of PPARγ agonists.

## Author Contributions

SM performed experiments, analyzed, and interpreted data, wrote the first drafts of the manuscript. PG and CD contributed to immunoblotting assays and analysis, and graphic presentation. SY helped with primary cultures and immunocytochemistry. SM, IS, and OA conceptualized the study. IS and OA supervised the work and finalized the manuscript. Parts of this work are adapted from a Ph.D. thesis by SM, submitted to Technical University, Munich (Ph.D. awarded: February 2019).

## Conflict of Interest Statement

The authors declare that the research was conducted in the absence of any commercial or financial relationships that could be construed as a potential conflict of interest.

## Abbreviations

Aβ: amyloid β
ABCA1: ATP-binding cassette transporter ABCA1
AD: Alzheimer’s disease
APP: amyloid precursor protein
BACE1: β-secretase 1
DIV: days *in vitro*
FCS: fetal calf serum
GFAP: glial acidic fibrillary protein [GFAP]
GluN2B: glutamatergic receptor 2B subunit
GSK-3β: glycogen synthase kinase 3β
PGC-1α: peroxisome proliferator-activated receptor gamma coactivator 1α
Pio: pioglitazone
PPARγ: peroxisome proliferator-activated receptor γ
PSD-95: postsynaptic density-95
p-Tau: phosphorylated tau
T2D: type 2 diabetes
TZD: thiazolidinediones.
