# Corrigendum: Activated PPARγ Abrogates Misprocessing of Amyloid Precursor Protein, Tau Missorting and Synaptotoxicity

**DOI:** 10.3389/fncel.2020.623279

**Published:** 2020-11-25

**Authors:** Susanne Moosecker, Patrícia Gomes, Chrysoula Dioli, Shuang Yu, Ioannis Sotiropoulos, Osborne F. X. Almeida

**Affiliations:** ^1^Department Stress Neurobiology and Neurogenetics, Max Planck Institute of Psychiatry, Munich, Germany; ^2^Graduate School, Technical University of Munich (TUM), Munich, Germany; ^3^Life and Health Sciences Research Institute (ICVS), Medical School, University of Minho, Braga, Portugal; ^4^ICVS/3B's – PT Government Associate Laboratory, Guimarães, Portugal

**Keywords:** Alzheimer's disease, amyloid beta, pioglitazone, PPARγ, neurons, Tau missorting, synaptic degradation

In the published article, there was an error regarding the affiliations for **Susanne Moosecker**. As well as having affiliation **1**, the author should also have **Graduate School, Technical University of Munich (TUM), Munich, Germany**.

The authors apologize for this error and state that this does not change the scientific conclusions of the article in any way. The original article has been updated.

