# Supplementary material for: Activated PPARγ Abrogates Misprocessing of Amyloid Precursor Protein, Tau Missorting and Synaptotoxicity
Source: Front Cell Neurosci. 2019 Jun 12;13:239. doi: 10.3389/fncel.2019.00239 (PMC6584807; doi:10.3389/fncel.2019.00239)
Supplement: Supplementary file 1 [file Data_Sheet_1.pdf]

Supplementary Figures

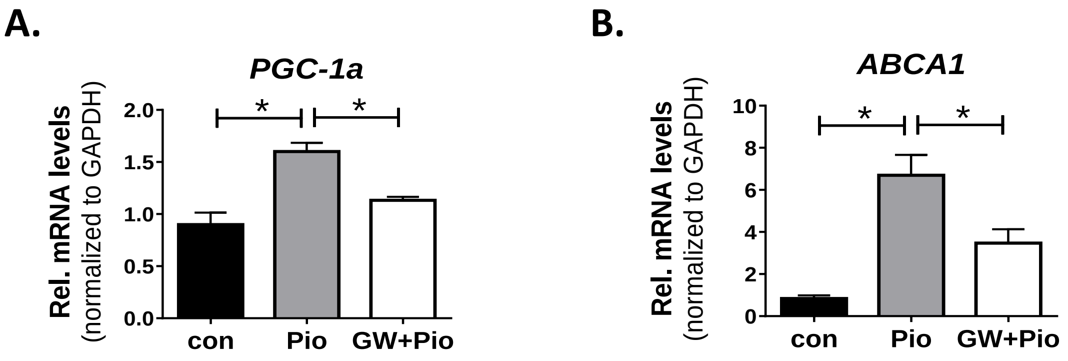

Supplementary Figure 1.
Pioglitazone (Pio) induced the expression of the mRNAs encoding two well-known PPARγ target genes, **(A)** *peroxisome proliferator-activated receptor gamma coactivator 1-alpha (PGC-1α)* and **(B)** *ATP-binding cassette transporter ABCA1 (ABCA1)*, in SHSY5Y neuroblastoma cells. The effects of Pio were blocked in the presence of the PPARγ antagonist GW9662. mRNA levels were determined by RT-PCR and normalized to *GAPDH* mRNA expression. Values shown are means ± S.E.M; significant differences are $p < 0.05$.

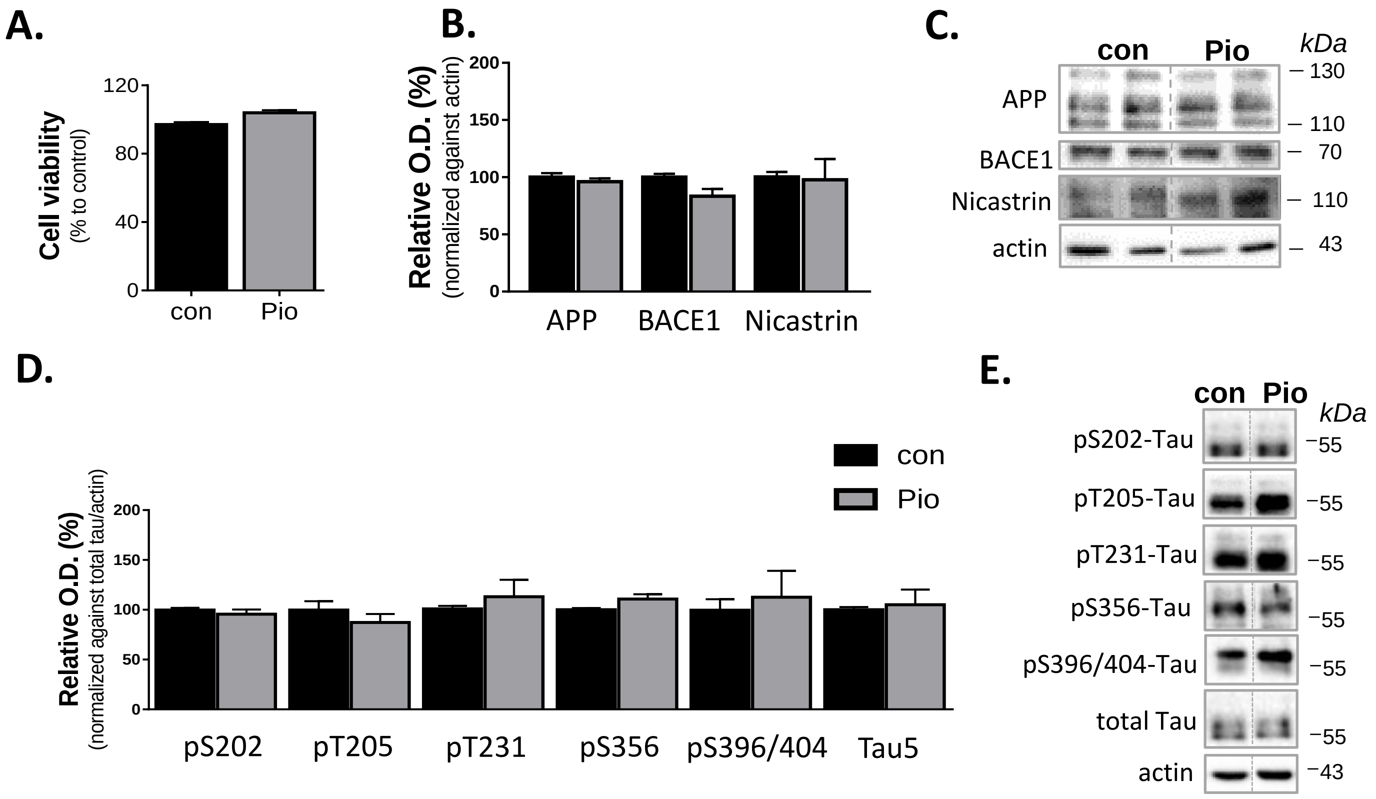

Supplementary Figure 2.

(A) Pioglitazone (Pio) alone did not alter the viability of SHSY5Y cells.

(B, C) Pio did not exert any effect on the expression of amyloid precursor protein (APP)or BACE1 and nicastrin protein expression in SHSY5Y cells under normal conditions.

(D, E) Immunoblot analysis revealed that the phosphorylation profile of Tau protein is not influenced when primary neurons are exposed to Pio alone. Numerical data shown are means ± S.E.M.

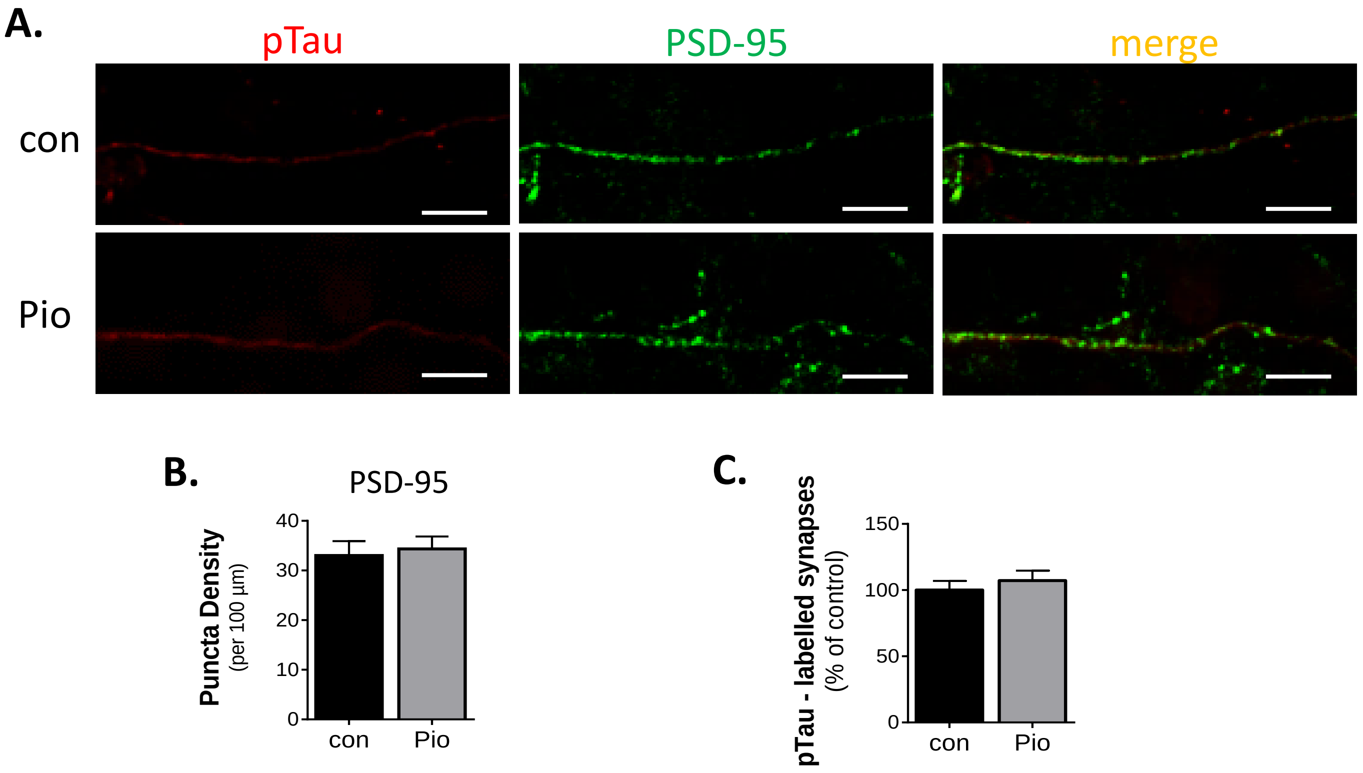

Supplementary Figure 3.

(A) Representative confocal images of primary neurons treated with vehicle or Pio demonstrate that Pio does not change synaptic puncta density; synapses are immunolabled with PSD-95 (*green*).

(B) Quantitative data that show that Pio does not influence PSD-95-positive synapses.

(C) Shows that Pio does not cause changes the percentage of pTau-positive synaptic puncta, i.e. Pio does not alter the amount of pTau localized in synapses under normal conditions.

Scale bar in **A**: 10 µm

Data shown in **B** and **C** are means ± S.E.M.
